# How to Sleep Well

**Published:** 1871-03

**Authors:** 


### HALL'S

### JOURNAL OF HEALTH.

Vol. XVIII. — MARCH, 1871. — No. III.

### HOW TO SLEEP WELL.

There can be no healthful sleep except that which follows the sleepiness resulting from the voluntary and involuntary action of the muscles of the human body. Weston, the great walker, falls into a sound, deep sleep almost as soon as he is put to bed, at the appointed time for rest. This is the sleep from voluntary muscular exercise. A person in good health sits around the house all day; an invalid may all day sit, and lounge, and lie down from morning until night, without sleeping; and both the healthy man and the invalid, in the course of the evening, will become sleepy, and fall into sound repose, the result of the weariness which involuntary motion brings about; for the various organs of the body, the heart, the liver, the stomach, the eyelids, work steadily every day. The intestines are as ceaseless in their motion as the waves of the ocean; as these latter are always dashing towards the shore, so is the great visceral machinery working, working, working, pushing the wastes of the body downwards and outwards from the first breath of existence to the last gasp of life.

There is not a movement of the system, voluntary or involuntary, external or internal, which does not require power to cause it. When that power is to a certain extent exhausted, instinct brings on the sensation of sleepiness, which is the result of exhausted power intended by nature to secure that cessation from active action which gives time for recuperation, very much as a man who runs for a while stops and rests, so as to get strength to run again.

We get up in the morning with a certain amount of reserve or accumulated strength; in the course of the day that strength becomes expended to the point necessary for the commencement of a new supply, which supply comes from rest, the rest of sleep.

Opium, narcotics, all forms of anodynes, cause sleep artificially, by compelling rest. A horse may be tied so that he cannot move ; he is compelled to be at rest; it is not the rest of tiredness, hence is unnatural. Anodynes in a sense tie a man down; they take away his power of motion, they compel a rest, but it is not the rest which is a result of used-up strength, hence it is an artificial rest, causing artificial sleep, not natural; and sleep which is not natural cannot be healthful; hence the truth of the first utterance of the chapter; healthful sleep comes from the expenditure of the strength of the body in various forms of exercise.

### SLEEPLESSNESS AND INSANITY.

There are more insane persons in the United States than in any other part of the world by one hundred per cent. " I can't sleep " is a complaint becoming every day more familiar to the city physician, and sleeplessness always precedes the ordinary forms of insanity ; on the other hand, an improvement in the ability to sleep is a certain indication of coming restoration to reason. Hence the want of ability to sleep well, soundly, and connectedly should always meet with prompt attention, to prevent its becoming a habit, a second nature. The speediest method of doing this is to break up the present associations, whatever may be the sacrifice ; get some different employment, something more active or stirring. The next best thing is a long journey on horseback, with a good companion ; a journey which has an end in view, the selection or location of lands for investment; camping out for months together as far as is practicable from human habitations, relying for provisions wholly on what can be caught or hunted. Visiting new and strange countries is another method of breaking up the treadmill sameness of some kinds of business. The great point for those who cannot sleep satisfactorily is to be a large portion of the time in the open air, and to be occupied in a way to bring into activity other muscles and other mental operations; and in proportion as they are of pleasurable and absorbing interest, so much the happier will be the good effect, and the more speedy will be the return to that "balmy sleep," the very thought of which, as enjoyed in youth, is a happiness. Neither money nor medicine can purchase healthful sleep ; it can only be procured in all its deliciousness by large outdoor activities or homely toil.

